# Supplementary material for: Beyond Information Provision: Analysis of the Roles of Structure and Agency in COVID-19 Vaccine Confidence in Ethnic Minority Communities
Source: Int J Environ Res Public Health. 2023 Nov 1;20(21):7008. doi: 10.3390/ijerph20217008 (PMC10650583; doi:10.3390/ijerph20217008)
Supplement: Supplementary file 1 [file ijerph-20-07008-s001.zip › File S1 Topic guide_professionals.pdf]

A qualitative study of reasons for reduced Covid-19 vaccine confidence in low uptake ethnic groups in London

[Before turning on the recorder]

- Introduce myself
- Go through the participant information sheet
- Go through consent form & sign
- Explain that I'm going to note down things I want to come back to
- Reiterate there's no right or wrong answer etc.

Introductions

- Can you tell me a bit about yourself?
  - What is you role
- Can you tell me about your work during Covid
  - Examples of community engagement
  - Which communities
  - Responses
  - Success/challenges

Concerns about vaccine

- Which concerns have you come across?
  - What other factors have influence views?
  - Gender
  - Age
  - Ethnicity
  - Religion
  - Where you live/work
  - Experience of healthcare
  - Family/friends
  - Ability to travel/vaccine passports
- Does having covid influence views?
- How have views changed over time?
- Impact of recent policy changes

Comparison with influenza vaccine

- Do you have any experience of working in other vaccine programmes?
- How does this compare?

Recommendations

- Who needs to give info about vaccines in future?
  - Government
  - NHS
  - Local GP/nurse/pharmacist
  - Friends
  - Family

    - Members of your community
- How can we make it easier for people to access trusted information?
    - Working with community groups
    - Online information
    - Government messaging

<u>Close</u>

That’s all I have to ask you but do you have any questions for me at all?

Thank you for your time
