# Supplementary material for: Beyond Information Provision: Analysis of the Roles of Structure and Agency in COVID-19 Vaccine Confidence in Ethnic Minority Communities
Source: Int J Environ Res Public Health. 2023 Nov 1;20(21):7008. doi: 10.3390/ijerph20217008 (PMC10650583; doi:10.3390/ijerph20217008)
Supplement: Supplementary file 1 [file ijerph-20-07008-s001.zip › File S2 The topic guide for members of the public.pdf]

<u>A qualitative study of reasons for reduced Covid-19 vaccine confidence in low uptake ethnic groups in London</u>

[Before turning on the recorder]

- Introduce myself
- Go through the participant information sheet
- Go through consent form & sign
- Explain that I'm going to note down things I want to come back to
- Reiterate there's no right or wrong answer etc.

<u>Introductions</u>

- Can you tell me a bit about yourself?
  - Where do you live?
  - What do you do?
  - Who do you live with?
- Can you tell me about your experiences during the Covid-19 pandemic/most memorable experience?
  - Have you or any of your family members been unwell with Covid?
  - Has it affected your work or living situation?
- When were you first offered a Covid vaccination?
- How did you make your decision?
  - Which sources of information did you access?
    - NHS website/GP/Practice nurse/social media/friends/family
- What was it like getting your vaccine?
  - Where did you get it?
  - Was it a positive experience?

<u>Concerns about vaccine</u>

- Can you tell me more about your concerns about the Covid vaccine?
  - These might include vaccine safety, effectiveness, side effects and long-term effects on health
- What other factors have influenced your views?
  - Gender
  - Age
  - Ethnicity
  - Religion
  - Where you live/work
  - Experience of healthcare
  - Family/friends
  - Ability to travel/vaccine passports
- Have you or anyone you know caught covid after having the vaccine and has this affected your views?
- Have your views of the vaccine changed over time and if so how?
- Has recent change in policy affected your decision (HCWs)?

Comparison with influenza vaccine

- Have you ever had a flu vaccine?
    - When did you last have it?
- How do you feel about flu vaccination, or other vaccines, and how does this compare to Covid vaccination?

Recommendations

- Who do you trust to give you accurate information about Covid vaccination?
    - Government
    - NHS
    - Local GP/nurse/pharmacist
    - Friends
    - Family
    - Members of your community
- How can we make it easier for people to access trusted information?
    - Working with community groups
    - Online information
    - Government messaging

Close

That’s all I have to ask you but do you have any questions for me at all?

Thank you for your time
